# Quality assurance of a gimbaled head swing verification using feature point tracking

**DOI:** 10.1002/acm2.12004

**Published:** 2016-11-21

**Authors:** Hideharu Miura, Shuichi Ozawa, Tsubasa Enosaki, Atsushi Kawakubo, Fumika Hosono, Kiyoshi Yamada, Yasushi Nagata

**Affiliations:** ^1^ Hiroshima High‐Precision Radiotherapy Cancer Center 3‐2‐2, Futabanosato Higashi‐ku Hiroshima 732‐0057 Japan; ^2^ Department of Radiation Oncology Institute of Biomedical and Health Science Hiroshima University 1‐2‐3, Kasumi, Minami‐ku Hiroshima 734‐8551 Japan

**Keywords:** computer processing, dynamic tumor tracking, quality assurance, Vero4DRT

## Abstract

To perform dynamic tumor tracking (DTT) for clinical applications safely and accurately, gimbaled head swing verification is important. We propose a quantitative gimbaled head swing verification method for daily quality assurance (QA), which uses feature point tracking and a web camera. The web camera was placed on a couch at the same position for every gimbaled head swing verification, and could move based on a determined input function (sinusoidal patterns; amplitude: ± 20 mm; cycle: 3 s) in the pan and tilt directions at isocenter plane. Two continuous images were then analyzed for each feature point using the pyramidal Lucas–Kanade (LK) method, which is an optical flow estimation algorithm. We used a tapped hole as a feature point of the gimbaled head. The period and amplitude were analyzed to acquire a quantitative gimbaled head swing value for daily QA. The mean ± SD of the period were 3.00 ± 0.03 (range: 3.00–3.07) s and 3.00 ± 0.02 (range: 3.00–3.07) s in the pan and tilt directions, respectively. The mean ± SD of the relative displacement were 19.7 ± 0.08 (range: 19.6–19.8) mm and 18.9 ± 0.2 (range: 18.4–19.5) mm in the pan and tilt directions, respectively. The gimbaled head swing was reliable for DTT. We propose a quantitative gimbaled head swing verification method for daily QA using the feature point tracking method and a web camera. Our method can quantitatively assess the gimbaled head swing for daily QA from baseline values, measured at the time of acceptance and commissioning.

## 1 Introduction

One of the most important issues faced when treating thoracic and abdominal cancer patients is intrafractional motion. The greatest movement is produced in the superior–inferior (SI) direction close to the diaphragm, such as with tumors in the lower lung lobes and upper abdominal tumors, such as liver or pancreatic tumors.^1^ Several methods have been proposed to compensate for respiratory‐induced organ motion, including forced shallow‐breathing, breath holding, respiratory gating, and dynamic tumor tracking (DTT).^1^, ^2^, ^3^, ^4^, ^5^, ^6^, ^7^, ^8^, ^9^, ^10^ DTT was realized through reasonably accurate real‐time acquisition of the target motion of a patient using external surrogates (indirect DTT) or an internally implanted marker (direct DTT). An overview of the management of respiratory motion in radiotherapy was summarized in the report of the American Association of Physicists in Medicine (AAPM) Task Group (TG) 76.^1^

The DTT techniques of the Vero4DRT™ system require synchronization of a gimbaled X‐ray head swing with the respiratory cycle of a patient, which is based on a surrogated infrared (IR) signal. The mass of the gimbaled head is approximately 600 kg.^2^ Thus, DTT with the Vero4DRT™ system may put additional stress on the gimbaled head, which may lead to unexpected changes in machine performance. The AAPM TG142 report includes a recommendation for general quality assurance (QA) tests for medical accelerators.^11^ Several investigators reported high tracking accuracies of DTT through the Vero4DRT™ system using video images and an Electronic Portal Imaging Device (EPID).^8^, ^9^, ^10^ Akimoto et al. proposed a useful QA method to readily assess the overall tracking accuracy using real‐time EPID images, which took only approximately 10 min, and for which analysis was performed each week when IR tracking was scheduled.^8^ Gimbaled head swing should be verified as a daily QA test, because patients come to a hospital to receive radiation therapy every day. From a mechanical perspective, it is necessary to visually confirm gimbaled head swing before clinical use. However, this test is only for verification of operation and is not quantitative; thus with this test the user cannot confirm whether the data deviate from baseline values.

To perform DTT safely and accurately for clinical purposes, gimbaled head swing verification is important. Here, we propose a quantitative gimbaled head swing verification method that uses feature point tracking and a web camera for daily QA.

## 2 Materials and methods

The Vero4DRT™ system (MHI‐TM2000; Mitsubishi Heavy Industries, Ltd., Tokyo, Japan, and BrainLAB, Feldkirchen, Germany) is described elsewhere.^6^, ^7^, ^8^ Briefly, the Vero4DRT™ system is equipped with a gimbaled head for DTT, a system‐specific fixed jaw, multileaf collimator (MLC), IR camera, and image‐guided radiotherapy (IGRT) system. The Vero4DRT™ system has a fixed primary collimator positioned upstream of the MLC without movable jaws. The gantry can be rotated ± 185° along an O‐shaped guideline at a nominal maximum speed of 7°/s, and the O‐ring can be rotated ± 60° around its vertical axis at a nominal maximum speed of 3°/s. The IR camera can monitor IR markers on the abdomen of a patient with high accuracy, and the IR marker position as well as a kilovoltage (kV) image can be used to perform 4D modeling. The Vero4DRT™ system is equipped with a dual orthogonal kV imaging system mounted on the ring at ± 45° on each side of the megavolt (MV) source, and kV cone‐beam computed tomography (CBCT) can be performed using each source–detector pair. The gimbaled head can swing along two orthogonal axes up to ± 2.5°. It swings a beam up to ± 41.9 mm in each direction with a maximum speed of 152 mm/s from the isocenter of the isocenter plane, allowing pan and tilt motion of the linear accelerator.

**Figure 1:**
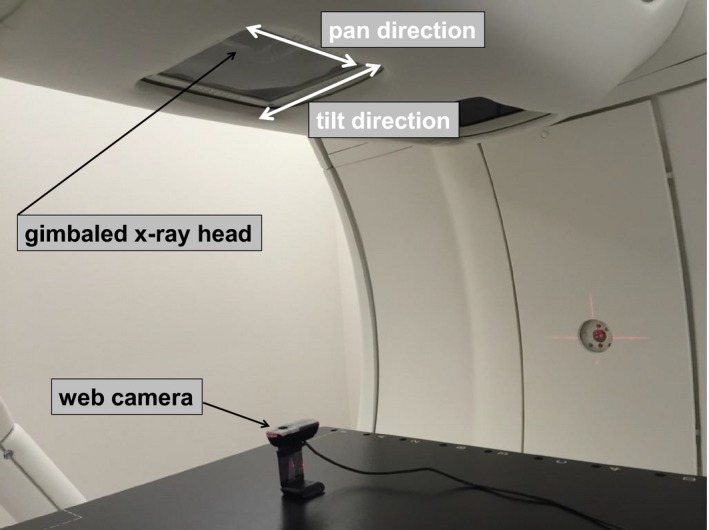
Experimental setup for gimbaled head swing verification. Web camera was placed on the couch at the same position for measurements. The pan and tilt motions correspond to beam swing in the left–right (LR) and superior–inferior (SI) directions, respectively.

**Figure 2:**
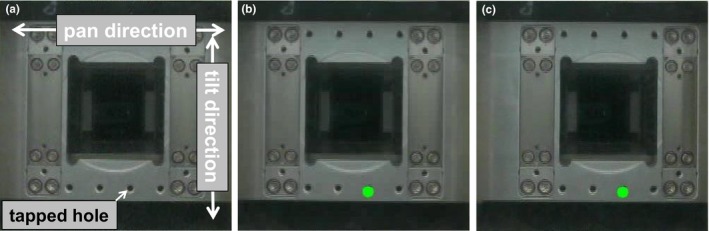
(a) This is an image using the web camera under the experiment setup. (b) The initialization points are in light green full circle on the tapped hole. (c) Initialized point was tracking along pan direction swing under featured‐point algorithm.

Figure 1 shows the experimental setup for gimbaled head swing verification for daily QA, which could move based on a determined input function (sinusoidal patterns; amplitude: ± 20 mm; cycle: 3 s) in the pan and tilt directions. A web camera was placed on a couch in the same position during the measurements. The web camera (Logicool C270, Logitech, Switzerland), capable of frame rates of up to 30 fps at a maximum resolution of 640 × 480 pixels, was connected to a computer via a USB interface. For quantitative gimbaled head swing verification, an in‐house software was developed using Microsoft Visual C++ and OpenCV (version 2.4.9), an open source image processing library.^12^ The web camera was used to record a swing of the gimbaled head. Figure 2 shows the procedure for gimbaled head swing verification using feature point tracking. Gimbaled head swing verification was carried out as follows: The initial position of the feature point and the initial video frame were initialized for the tracking process. We used a tapped hole as a feature point of the gimbaled head. Two continuous images were then analyzed for each feature point using the pyramidal Lucas–Kanade (LK) method, which is an optical flow estimation algorithm.^13^ Optical flow is the pattern of apparent motion of image objects between two consecutive frames caused by the movement of object or camera. The optical flow methods try to calculate the motion between two image frames which are taken at times *t* and *t* + δ*t* at every pixel position.^14^ This estimation was based on two assumptions; first, the intensity of any object point is constant over time and second, nearby points on the image plane follow a similar flow.^15^ The algorithm calculates the coordinates of the feature points in the current video frame given their coordinates in the previous frame. The difference between peak and valley values from the wave was the gimbaled head swing, defined as “amplitude”. The difference between consecutive peak (valley) and peak (valley) temporal values of the wave was defined as “period”. We performed the measurement at a gantry angle of 0° to investigate the quantitative gimbaled head swing verification in the pan and tilt directions. Five waves were analyzed for daily QA; thus, we used frame images of 15 s. In this study, gimbaled head swing verification was reported for 30 consecutive days (except for weekends and holidays).

## 3 Results

**Figure 3:**
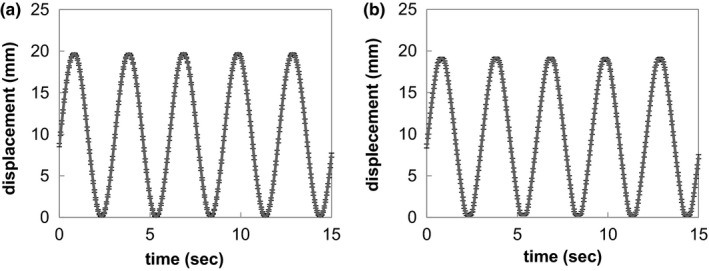
Displacement of gimbaled head swing along (a) pan and (b) tilt directions measured as the average and standard deviation. The data were collected consecutively for 30 d. The error bars correspond to the standard deviation.

Figure 3 shows the gimbaled head swing amplitude as a function of time using feature point tracking. The mean ± SD of the period was 3.00 ± 0.03 (range: 3.00–3.07) s and 3.00 ± 0.02 (range, 3.00–3.07) s in the pan and tilt directions, respectively. The mean ± SD of the relative displacement was 19.7 ± 0.08 (range: 19.6–19.8) mm and 18.9 ± 0.2 (range: 18.4–19.5) in the pan and tilt directions, respectively. This QA method took approximately 10 min, including setting the web camera, recording gimbaled head swing, and analyzing the data.

## 4 Discussion

In this study, our proposed method was used to verify the quantitative gimbaled head swing for daily QA using feature point tracking and a web camera. Most users usually cannot evaluate the quantitative value from gimbaled head swing verification. Thus, there may be unexpected changes in machine performance due to machine malfunction, mechanical breakdown, physical accidents, or component failure. In addition, there may be gradual changes as a result of aging of the machine components. These patterns of failure must be considered when establishing a periodic QA program. We demonstrated that the gimbaled head swing verification can be performed at frequent intervals.

We know that gimbaled head swing verification has an amplitude of ± 20 mm and a period of 3 s in the pan and tilt directions at isocenter plane. This information was obtained by personal communication with the vender. As for the period, our result for the period was slightly different from the vender information. This may be attributed to the frame rate limitation, not to mechanical error. The spatial resolution of the web camera with an image size of 640 × 480 pixels on the gimbaled head was approximately 0.5 mm in our study. Therefore, there is a possibility of the spatial resolution limitations in feature point tracking.

The mean amplitude value for the tilt direction was slightly smaller than that of the pan direction, because the image viewed from the web camera was slightly altered by the swing of the gimbaled head. To eliminate this discrepancy, the feature point should be placed at the center of gimbaled head. However, this is difficult to achieve because the aperture is located at the center of the gimbaled head. Thus, we placed the feature point at the same point for every verification. Other features point such as bolts can be used for tracking. However, same feature point always should be used to compare the data between measured and baseline values. Unfortunately, we cannot compare deviation from baseline value at the time of acceptance and commissioning, because gimbaled head swing verification using our proposed method had not been established. However, we acquired the baseline value of our proposed method when we measured the tracking accuracy using a film, based on 4D‐modeling using a surrogate signal.^5^

One limitation of this study is that it only investigated short‐term gimbaled head swing verification. Further studies should examine the long‐term stability of gimbaled head swing verification for daily QA. The Vero4DRT™ system is used for DTT based on an external surrogate signal. Our proposed method cannot evaluate the overall tracking accuracy based on 4D‐modeling using a surrogate signal. Our proposed method has the advantage that it can be used to confirm, quite efficiently and quantitatively, the gimbaled head swing verification, even immediately before treatment.

## 5 Conclusions

We proposed a quantitative gimbaled head swing verification method for daily QA using a feature point tracking method and web camera. From our results, the gimbaled head swing was reliable for DTT. Our method can assess the quantitative gimbaled head swing from baseline values, measured at the time of acceptance and commissioning.
